# Supplementary material for: Comprehensive detection of structural variation and transposable element differences between wild type laboratory lineages of C. elegans
Source: bioRxiv. 2023 Nov 3:2023.01.13.523974. Preprint. [Version 2] doi: 10.1101/2023.01.13.523974 (PMC10634987; doi:10.1101/2023.01.13.523974)
Supplement: 1 [file NIHPP2023.01.13.523974V2-supplement-1.pdf]

bioRxiv preprint doi: https://doi.org/10.1101/2023.01.13.523974; this version posted November 3, 2023. The copyright holder for this preprint (which was not certified by peer review) is the author/funder, who has granted bioRxiv a license to display the preprint in perpetuity. It is made available under aCC-BY 4.0 International license.

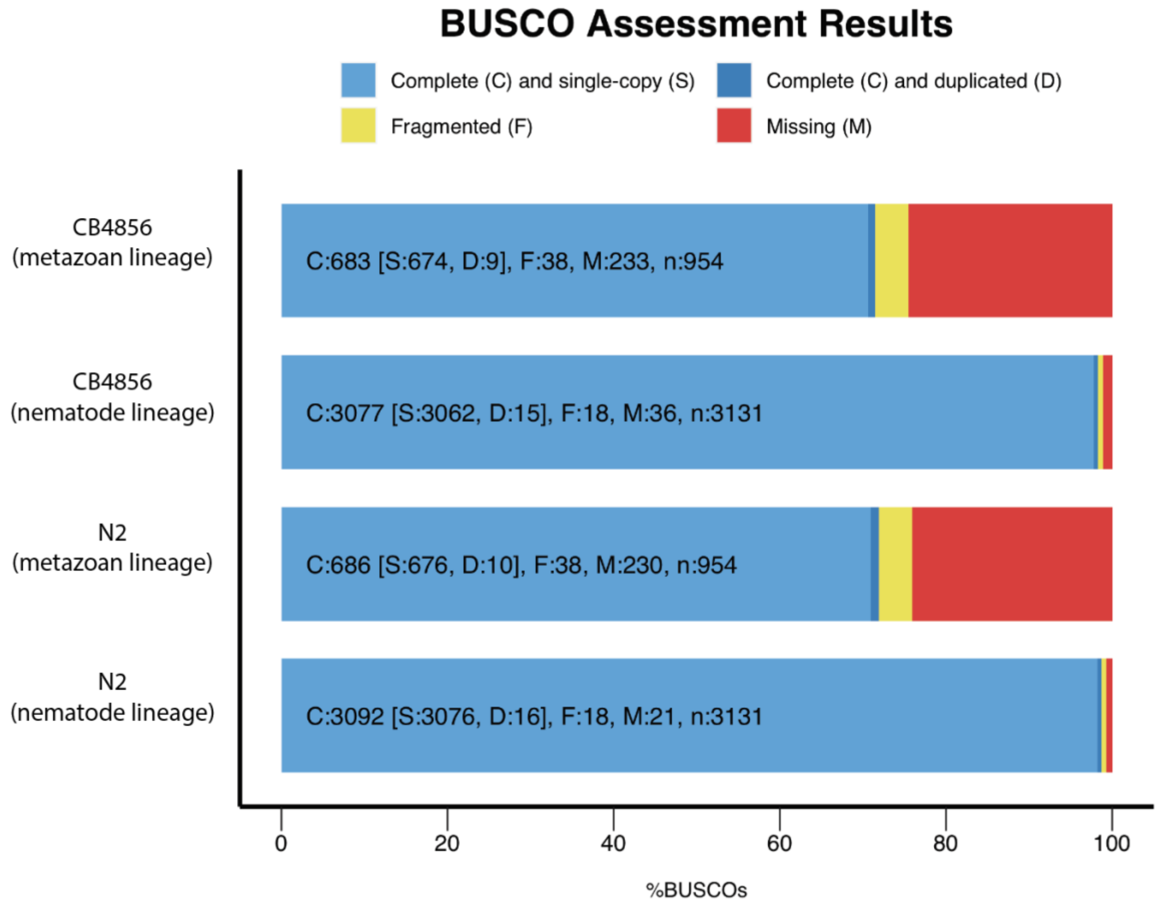

Supplemental Figure S1. BUSCO analysis of the DLW N2 Bristol and DLW CB4856 Hawaiian genome assemblies. The presence of orthologous genes from metazoan and nematode lineages are shown for each genome assembly. Each orthologous gene analyzed is depicted as either Complete (C, blues), Fragmented (F, yellow), or Missing (M, red). Complete orthologs are then further categorized as single-copy (S, light blue) or duplicated (D, dark blue).

bioRxiv preprint doi: https://doi.org/10.1101/2023.01.13.523974; this version posted November 3, 2023. The copyright holder for this preprint (which was not certified by peer review) is the author/funder, who has granted bioRxiv a license to display the preprint in perpetuity. It is made available under aCC-BY 4.0 International license.

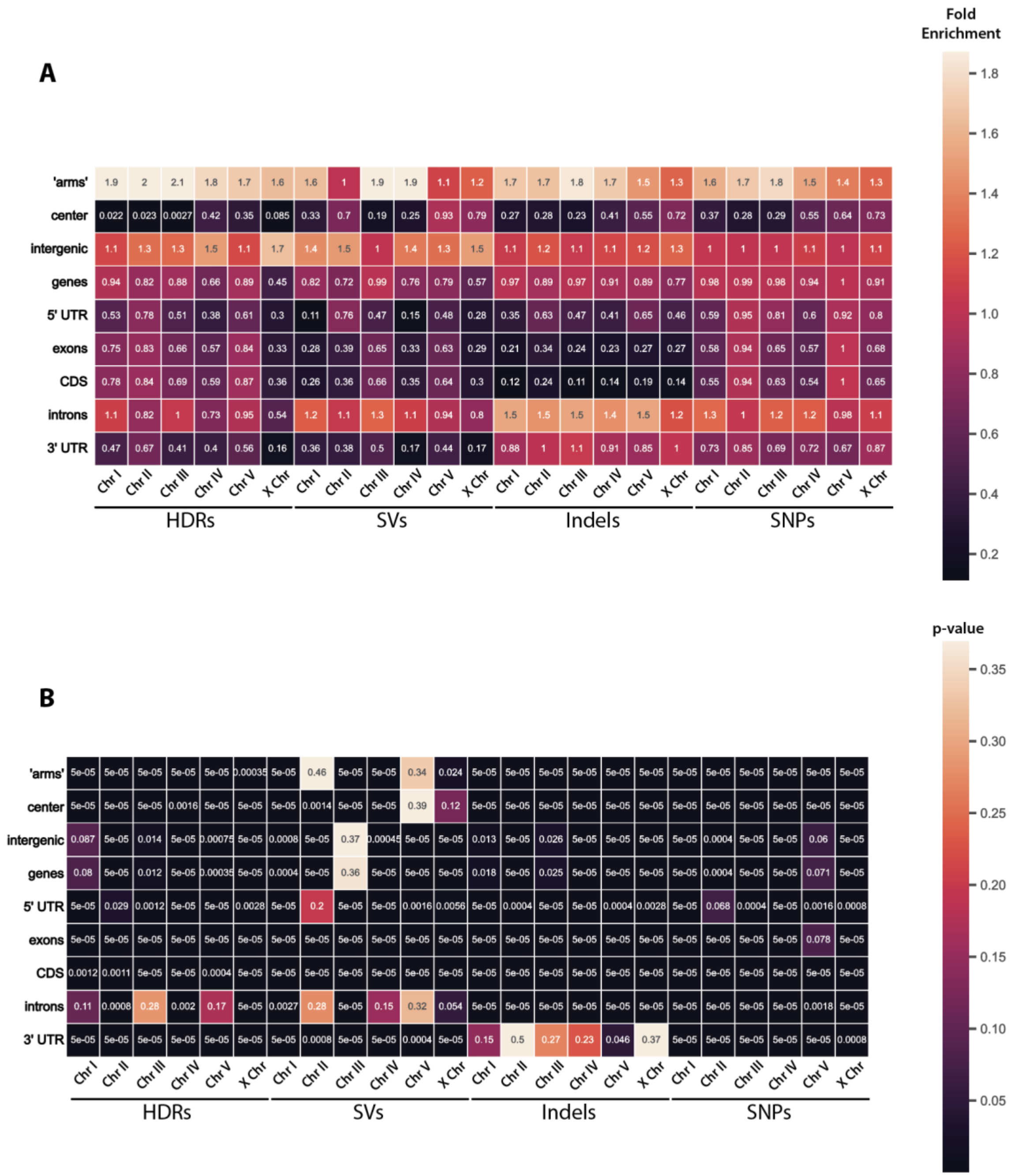

Supplemental Figure S2. GAT interval-association test results analyzing the overlap of DLW CB4856 Hawaiian SNPs, indels, and SVs with N2 genome annotations. A) Heatmap showing the fold enrichment of each variant type within gene annotations for each chromosome. B) Heatmap of p-values associated with corresponding fold enrichments shown in panel A calculated by the hypergeometric test.

bioRxiv preprint doi: https://doi.org/10.1101/2023.01.13.523974; this version posted November 3, 2023. The copyright holder for this preprint (which was not certified by peer review) is the author/funder, who has granted bioRxiv a license to display the preprint in perpetuity. It is made available under aCC-BY 4.0 International license.

Supplemental Table 1. Transposable Elements identified in DLW N2 Bristol genome (this study) vs DLW CB4856 Hawaiian genome (this study)

	DLW N2 Bristol	DLW CB4856 Hawaiian
**Class I Transposable Elements (Retrotransposons)**	710 (2,688,730 bp)	776 (2,522,357 bp)
**Gypsy**	557 (2,195,895 bp)	592 (2,031,038 bp)
**Copia**	134 (472,195 bp)	161 (465,785 bp)
**SINE**	9 (2,146 bp)	9 (1,945 bp)
**ERV**	7 (8,280 bp)	6 (7,569 bp)
**LINE**	3 (10,214 bp)	8 (16,038 bp)
**Class I intrachromosomal transpositions***	0	
**Class I interchromosomal transpositions***	0	
**Class II Transposable Elements (DNA transposons)**	17,682 (12,055,357 bp)	17,310 (11,606,010 bp)
**Tc1/Mariner**	1870 (1,298,386 bp)	1,550 (1,131,443 bp)
**hAT**	3,999 (3,988,461 bp)	3,818 (3,725,667 bp)
**CMC**	1,679 (3,138,647 bp)	2,011 (3,260,455 bp)
**Zator**	9,159 (3,009,341 bp)	8,980 (2,907,391 bp)
**Novosib**	46 (12,060 bp)	28 (12,088 bp)
**Helitron**	39 (368,980 bp)	43 (329,238 bp)
**Sola**	821 (226,645 bp)	699 (196,797 bp)
**MITE**	69 (12,837 bp)	181 (42,931 bp)
**Class II intrachromosomal transpositions***	38	
**Class II interchromosomal transpositions***	9	

* All TE sequences with predicted transpositions are relative to the DLW N2 Bristol genome

bioRxiv preprint doi: https://doi.org/10.1101/2023.01.13.523974; this version posted November 3, 2023. The copyright holder for this preprint (which was not certified by peer review) is the author/funder, who has granted bioRxiv a license to display the preprint in perpetuity. It is made available under aCC-BY 4.0 International license.

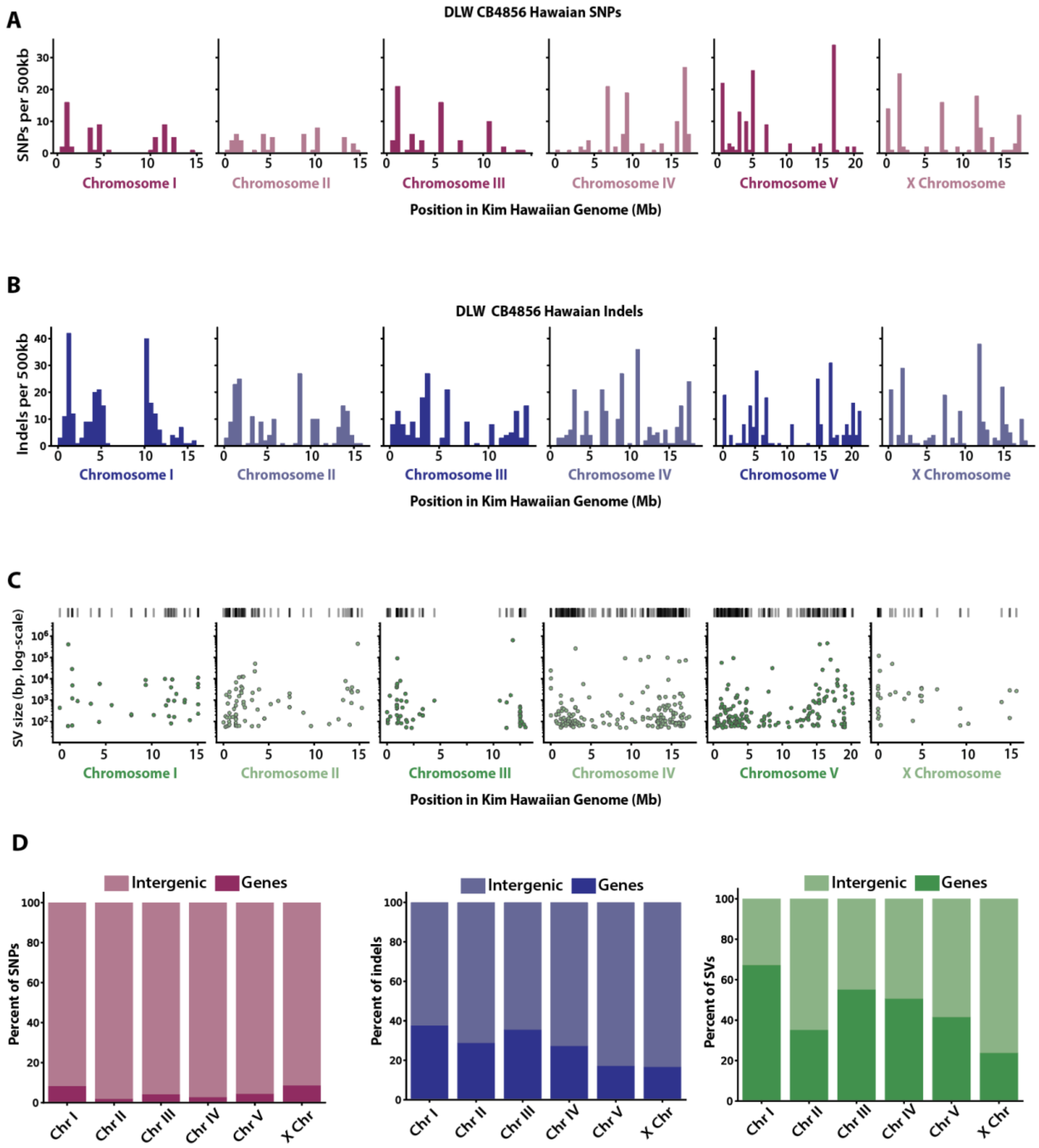

bioRxiv preprint doi: https://doi.org/10.1101/2023.01.13.523974; this version posted November 3, 2023. The copyright holder for this preprint (which was not certified by peer review) is the author/funder, who has granted bioRxiv a license to display the preprint in perpetuity. It is made available under aCC-BY 4.0 International license.

Supplementary Figure S3. Genomic variation between the DLW CB4856 Hawaiian genome and the Kim CB4856 Hawaiian genome. (A-B) Histograms depicting the distribution of SNPs and indels across each Kim CB4856 Hawaiian chromosome in 500kb bins. (C) Scatterplots showing the genomic position of SVs with the log-scaled size of each SV on the y-axis. (D) The proportions of DLW CB4856 Hawaiian SNPs, indels, and SVs that overlap with intergenic versus gene-coding regions of the Kim CB4856 Hawaiian genome.

bioRxiv preprint doi: https://doi.org/10.1101/2023.01.13.523974; this version posted November 3, 2023. The copyright holder for this preprint (which was not certified by peer review) is the author/funder, who has granted bioRxiv a license to display the preprint in perpetuity. It is made available under aCC-BY 4.0 International license.

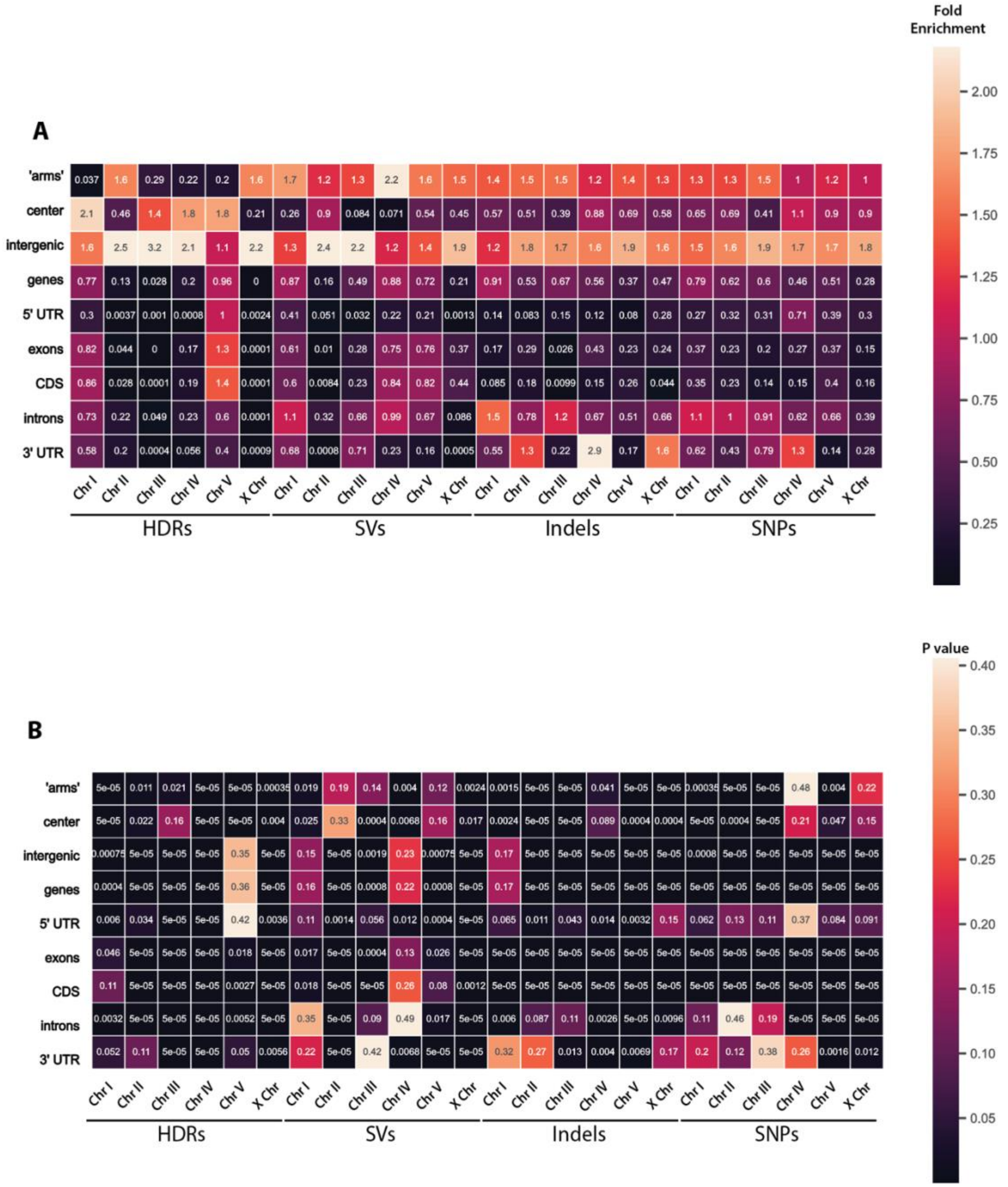

Supplemental Figure S4. GAT interval-association test results analyzing the overlap of DLW N2 Bristol SNPs, indels, and SVs with remapped VC2010 Bristol genome annotations. A) Heatmap showing the fold enrichment of each variant type within gene annotations for each

bioRxiv preprint doi: https://doi.org/10.1101/2023.01.13.523974; this version posted November 3, 2023. The copyright holder for this preprint (which was not certified by peer review) is the author/funder, who has granted bioRxiv a license to display the preprint in perpetuity. It is made available under aCC-BY 4.0 International license.

chromosome. B) Heatmap of p-values associated with corresponding fold enrichments shown in panel A calculated by the hypergeometric test.

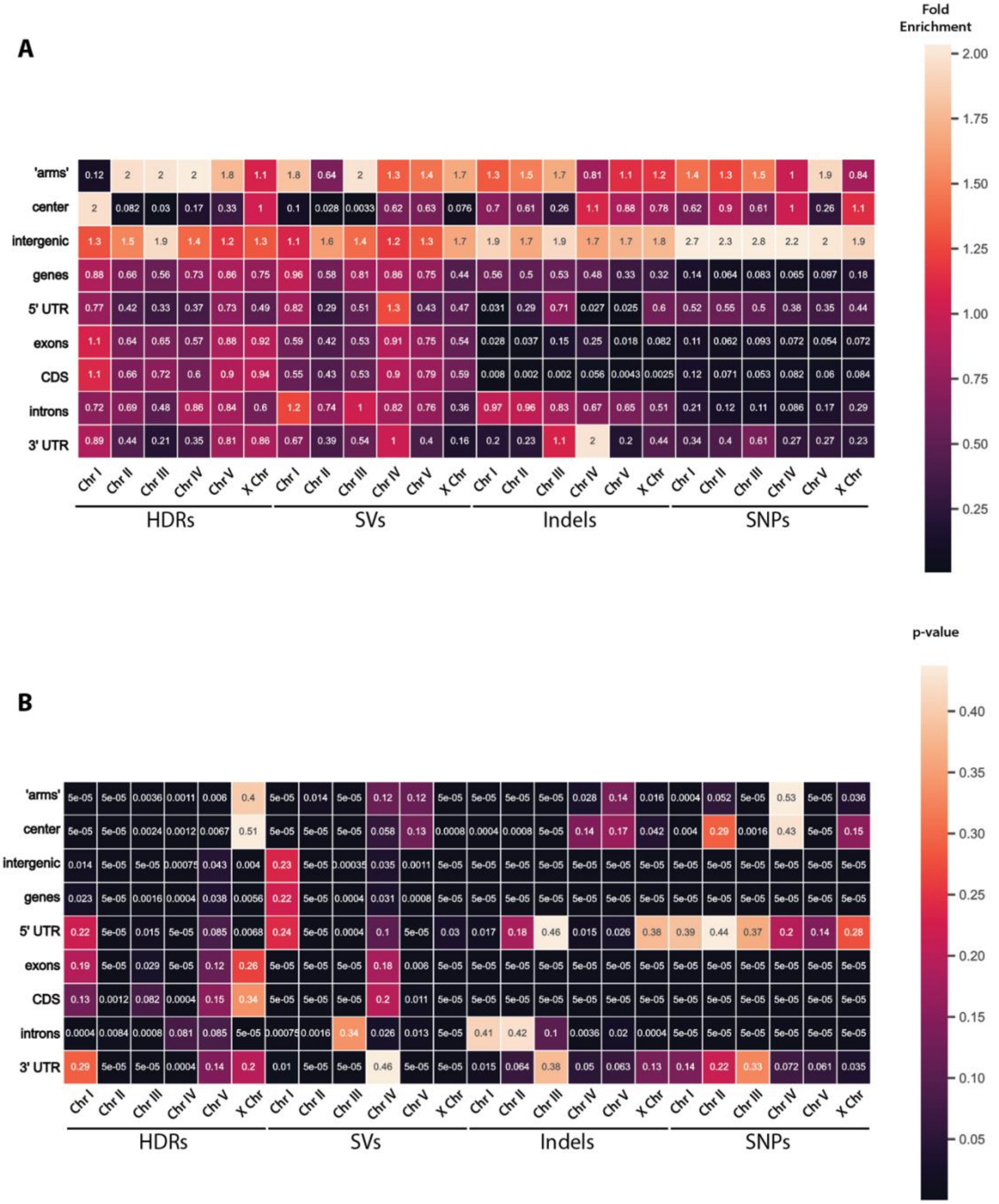

Supplemental Figure S5. GAT interval-association test results analyzing the overlap of DLW CB4856 Hawaiian SNPs, indels, and SVs with remapped Kim CB4856 Hawaiian genome annotations. A) Heatmap showing the fold enrichment of each variant type within gene annotations for each chromosome. B) Heatmap of p-values associated with corresponding fold enrichments shown in panel A calculated by the hypergeometric test.
